# Habitual coffee consumption and risk of frailty in later life: the Longitudinal Aging Study Amsterdam (LASA)

**DOI:** 10.1007/s00394-025-03683-0

**Published:** 2025-04-24

**Authors:** Mette van der Linden, Hanneke A.H. Wijnhoven, Laura A. Schaap, Emiel O. Hoogendijk, Margreet R. Olthof

**Affiliations:** 1 Department of Health Sciences, Faculty of Science, Amsterdam Public Health Research Institute, Vrije Universiteit Amsterdam, Amsterdam, 1081 HV the Netherlands; 2 Amsterdam Movement Sciences, Amsterdam, the Netherlands; 3 Department of Epidemiology & Data Science, Amsterdam Public Health Research Institute, Amsterdam UMC - location VU University Medical Center, Amsterdam,, the Netherlands; 4 Department of General Practice, Amsterdam Public Health Research Institute, Amsterdam UMC– location VU University Medical Center, Amsterdam, the Netherlands

**Keywords:** Coffee consumption, Frailty phenotype, Older adults, Observational study, Epidemiology

## Abstract

This study examined associations of current habitual and midlife coffee consumption with risk of (pre-)frailty in 1161 community-dwelling older adults (≥ 55 years) participating in the Longitudinal Aging Study Amsterdam.

Habitual and retrospectively assessed midlife (ages 40–65) coffee consumption was measured using questionnaires and divided into five categories (no coffee, > 0–2, > 2–4, > 4–6, > 6 cups/day). Frailty status was assessed using Fried’s five-component frailty phenotype. Generalized estimating equations, Cox proportional hazards models, and logistic regression analyses were performed to assess the associations of coffee consumption with frailty and pre-frailty prevalence, and the 3- and 7-year incidence of (pre)frailty.

Habitual coffee consumption of > 4–6 and > 6 cups/day was associated with lower odds of frailty compared with consumption of > 0–2 cups/day (ORs (95%CI) of 0.36 (0.16–0.82) and 0.37 (0.16–0.84), respectively). Similar but statistically non-significant associations were found for coffee consumption during midlife and between habitual coffee consumption and the 3- and 7-year incidence of frailty, except for a statistically significant lower hazard (HR: 0.41 [95%CI 0.23–0.71]) of frailty after 7 years for the consumption of > 2–4 cups/day compared to > 0–2 cups/day. No associations were found between coffee consumption and pre-frailty, with the exception of lower odds for those who consumed > 2–4 cups/day compared to > 0–2 cups/day (OR 0.73 [95%CI 0.54–0.99])

The results of this study indicate that higher habitual coffee consumption is associated with lower odds of frailty. Further research is needed to confirm our findings and investigate possible underlying mechanisms by which coffee might influence frailty development.

**Supplementary Information:**

The online version contains supplementary material available at 10.1007/s00394-025-03683-0.

## Introduction

Frailty is a complex geriatric condition that is characterized by a decline in multiple physiological systems, which increases the risk of adverse health outcomes such as disability, hospitalization, and death [1]. As the global population ages and the number of older adults increases, frailty will become an increasingly important issue in society [2]. The condition imposes both individual and societal burdens, such as a lower quality of life [3], loneliness [4], and an increased demand for long-term care [5].

Diet is considered an important determinant of frailty development in older adults [6, 7]. Studies have shown that nutritional interventions aimed at improving diet quality can help prevent or delay the onset of frailty [8]. Coffee is a common dietary component that has been associated with various health benefits, including a reduced risk of type 2 diabetes, certain types of cancer, cardiovascular disease, and reduced mortality [9, 10]. The health benefits associated with coffee consumption are often attributed to the presence of various bioactive compounds in coffee, such as caffeine and polyphenols [11], which have antioxidant and anti-inflammatory properties [12, 13]. Considering the involvement of inflammation and neuroendocrine dysregulation in the pathophysiology of frailty [14], coffee consumption may also be linked to a lower risk of frailty.

However, research on the association between coffee consumption and frailty is inconsistent, and the longitudinal relationship between coffee consumption and the risk of frailty has not been studied frequently. Four observational studies, including three cross-sectional studies [15–17] and one longitudinal study with a 20-year follow-up [18], found that higher coffee consumption [15, 16, 18] and caffeinated, but not decaffeinated coffee consumption [17] was associated with lower odds of frailty. In contrast, another longitudinal study, with a 7.2-year follow-up, did not find an association between coffee consumption and frailty [19]. Additionally, no significant association was found between genetically predicted coffee consumption and the risk of frailty in a Mendelian randomization study [20].

In conclusion, the association between coffee consumption and the risk of frailty remains uncertain. Data from the Longitudinal Aging Study Amsterdam (LASA) provide a valuable opportunity to investigate this relationship comprehensively, complementing the existing evidence. Therefore, this study examined the associations of habitual coffee consumption with frailty and pre-frailty prevalence, as well as the 3- and 7-year incidence of (pre-)frailty in Dutch community-dwelling older adults participating in LASA. In addition, we investigated whether retrospectively assessed coffee consumption during midlife (ages 40–65) is associated with frailty or pre-frailty in later life. As secondary analyses, we investigated associations of coffee consumption with individual components of frailty, and we investigated whether the presence of caffeine in coffee moderates the association between coffee consumption and frailty.

## Methods

### Study design and population

Data were used from the Longitudinal Aging Study Amsterdam (LASA). LASA is a large ongoing cohort study designed to investigate the physical, psychological, and social aspects of aging in a large representative sample of older adults in the Netherlands [21]. Data collection started in 1992/1993 among a cohort of adults aged 55–85 years old (*N* = 3107, wave B, baseline). Since then, measurement cycles have been conducted roughly every three years. The study sample was drawn from a variety of municipalities across three culturally distinct regions in the Netherlands. In 2002/2003 and 2012/2013, a second and a third cohort of adults aged 55 to 65 years were added to the original sample. Data collection includes a structured general interview, a structured medical interview with clinical measurements and a self-administered questionnaire. Further information on sampling and data collection can be found elsewhere [21–23].

**Fig. 1:**
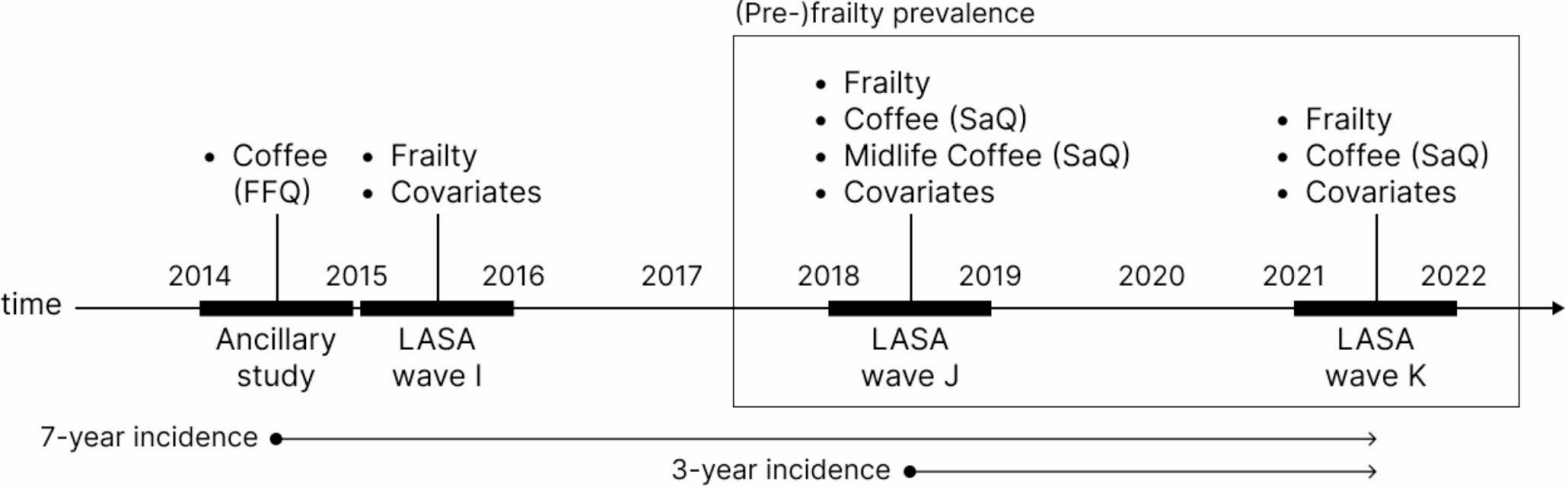
Timeline of data used for the current study

Figure 1 depicts the data utilized in the current study over time. To analyze associations of habitual coffee consumption and frailty or pre-frailty prevalence, as well as the 3-year incidence, we used data on habitual coffee consumption, frailty status, and covariates from the two most recent LASA waves, collected in 2018/2019 (wave J) and 2021/2022 (wave K). These waves provided the most detailed information on coffee consumption, assessed by a self-administered questionnaire (SaQ). For analyses of coffee consumption during midlife (ages 40–65) and frailty status in later life, we used data on midlife coffee consumption, retrospectively assessed by a SaQ in 2018/2019 (wave J). To analyze associations between habitual coffee consumption and the 7-year incidence of (pre-)frailty, we used data on coffee consumption from an ancillary study (*N* = 1439) conducted in 2014/2015. In this ancillary study, coffee consumption was assessed using a food frequency questionnaire (FFQ) [24]. Baseline data on frailty status and covariates were collected during the third most recent LASA wave in 2015/2016 (wave I). Follow-up data on frailty status were collected in 2018/2019 (wave J) and 2021/2022 (wave K). Ethical approval for the LASA study and the side study was given by the Medical Ethics Committee of the VU University Medical Center Amsterdam. All participants provided written informed consent.

### Frailty assessment

Frailty status was determined using Fried’s five-component frailty phenotype [1], one of the most commonly used and widely accepted operational definitions of frailty [25]. The frailty phenotype, as defined by Fried et al. [1], is a clinical syndrome characterized by the presence of three or more of the following criteria: weight loss, weakness, exhaustion, slow gait speed, and low physical activity. In LASA, the measures and cut-off values for these criteria were identical or similar to those used by Fried and colleagues [1]. For those measures that were not identical (gait speed and physical activity), the lowest quintile approach was used [26]. This approach has been applied successfully in previous studies with LASA data [27–29].

Weight loss was considered present if a participant had lost 5% or more of their body weight over the past three years (i.e. measured body weight at a given LASA measurement wave compared to the previous wave). Weight was measured in underwear to the nearest 0.1 kilogram (kg), using a calibrated bathroom scale (Seca, model 100, Lameris, Utrecht, the Netherlands) [30]. Weakness was operationalized as grip strength and measured using a hand-held dynamometer, taking the sum of the highest values of two measurements on each hand. Participants were assessed in a standing position with the elbow extended, or in a sitting position if standing was not possible. The original cut-off points by Fried et al. [1], stratified by sex and body mass index (BMI), were used to indicate poor grip strength. Two items from the Centre for Epidemiologic Studies Depression Scale (CES-D) were used to determine exhaustion. Exhaustion was considered present if a participant answered ‘often’ or ‘most of the time’ to the following two statements: “In the past week, I felt that everything I did was an effort” and “In the past week, I could not get going”. Physical activity was measured using the validated LASA Physical Activity Questionnaire; an interviewer-administered questionnaire that estimates the frequency and duration of participation in activities over the past 2 weeks [31]. Low physical activity was determined by the lowest quintile of the average time spent on walking and cycling per day in the past two weeks. Finally, gait speed was assessed by measuring the time (in seconds) it took participants to walk 3 m, make a turn, and then walk the same distance back quickly as possible. Slow gait speed was determined based on the lowest quintile, stratified by sex and height. Participants were classified as “robust” (0 components present), “pre-frail” (1–2 components present), or “frail” (3–5 components present). A maximum of two missing frailty components were permitted [1].

### Assessment of coffee consumption

In 2018/2019 and in 2021/2022 participants reported their coffee consumption in the past month by indicating the frequency of consumption in days per week (none; < 1; 1; 2; 3; 4; 5; 6; 7 days) and the number of cups consumed on these days (1; 2; 3; 4; 5; 6; 7; 8; 9; ≥10 cups) for each type of coffee (caffeinated; decaffeinated). Participants also reported the coffee cup volume. Response options were ‘small (about 125 ml)’, ‘medium (about 165 ml)’, ‘large (about 225 ml)’, ‘other, namely… ml’, and ‘don’t know’. If the cup size was unknown (don’t know) or missing, the value was imputed with the mean cup volume in the data (160 mL). In 2018/2019, participants older than 65 years were also asked about their coffee consumption during midlife (ages 40–65), using the same questions. From this information we calculated the average coffee consumption (habitual and midlife) in standardized cups of 125 ml per day [32]. Individuals were also categorized based on the type of coffee they consumed (caffeinated only; decaffeinated only; both caffeinated and decaffeinated). In the 2014/2015 ancillary study, participants reported the frequency of coffee consumption in the past four weeks (none; 1 day in the past four weeks; 2–3 days in the past four weeks; 1 day/week; 2 days/week; 3 days/week; 4 days/week; 5 days/week; 6 days/week; 7 days/week) as well as the number of cups they consumed on days of consumption (< 1; 1; 2; 3; 4; 5; 6; 7; 8; 9; 10; 11; 12; >12 cups). Based on this information, we calculated the average habitual coffee consumption in standardized cups of 125 mL per day.

For data analyses, coffee consumption was categorized into five categories: 0 cups/day, > 0–2 cups/day, > 2–4 cups/day, > 4–6 cups/day, and > 6 cups/day. This was done to improve comparability between studies, facilitate interpretation of the results, and to reflect the association with (pre-)frailty more accurately, as there was no clear linear relationship between the variables. Participants who consumed > 0–2 cups of coffee per day were used as the reference group in the analyses due to the low number of non-coffee consumers (< 5% of participants). Using a larger reference group can increase statistical power, reduce variability, and improve the generalizability and precision of estimates, resulting in more reliable and robust results. Despite the low representation of non-coffee consumers, they were analyzed as a distinct group since this group may have specific characteristics for abstaining from coffee consumption [33] and are therefore less suitable as a reference group. Lastly, it is important to note that, depending on the type of coffee consumed and the method of preparation, individuals in the highest categories of coffee consumption (i.e. >4–6 cups/day and > 6 cups/day) may exceed the daily caffeine limit of 400 mg, as recommended by the European Food Safety Authority (EFSA) [34].

### Assessment and operationalization of covariates

Information on age, sex, education, partner status, smoking status, alcohol use, tea consumption, BMI, number of chronic diseases, depressive symptoms, cognitive function, and sleep duration was collected through structured interviews and self-administered questionnaires during regular LASA measurement waves. Level of attained education was categorized into three groups: low (elementary not completed; elementary education; lower vocational education), intermediate (general intermediate education; intermediate vocational education; general secondary education), and high (higher vocational education; college education; university education). Partner status was categorized as ‘living alone’ and ‘living with partner’. Smoking status was determined by self-report of current and past cigarette smoking (yes/no); participants were categorized as ‘never smoked’, ‘former smoker’ or ‘current smoker’. Alcohol use was assessed during the structured medical interview with a questionnaire developed by Statistics Netherlands [35]. Using a classification system developed by the Netherlands Economic Institute, participants were categorized into ‘no use,’ ‘moderate use,’ ‘gray area,’ and ‘excessive use,’ adjusted for sex. Because of a low number of participants in the ‘excessive use’ group, we combined it with the ‘grey area’ group, creating a combined category labeled as ‘above moderate use’. Tea consumption was assessed in the FFQ of the ancillary study and with a self-administered questionnaire in 2018/2019 and 2021/2022. In both questionnaires, the questions asked were identical to those regarding coffee consumption. With this information, average daily tea consumption was calculated and categorized as ‘0 cups’, ‘>0–2 cups’, ‘>2–4 cups’ and ‘>4 cups’. To account for missing values of tea consumption (> 5% missing data) in 2018/2019 and 2021/2022, an extra category was added consisting of participants with missing data on tea consumption. BMI was calculated as weight (kilograms)/height (meters)^2^. Height was measured to the nearest 0.001 meter using a stadiometer. Participants were asked about the presence of chronic nonspecific lung disease, cardiac disease, peripheral arterial disease, stroke, diabetes mellitus, arthritis, and malignancies. From this information, a continuous variable representing the number of chronic diseases was constructed, ranging from zero to seven. Depressive symptoms were measured with the CES-D [36]. The CES-D scale ranges from zero to 60, with higher scores indicating a greater presence of depressive symptoms. The variable was dichotomized using a cut-off value of 16 or higher to identify participants with clinically relevant symptoms of depression [37, 38]. Cognitive function was assessed using a combined version of the Mini-Mental State Examination (MMSE), incorporating the highest scores from both subtraction and spelling tasks. The variable was dichotomized using a cut-off value of 23 or lower to identify participants with cognitive impairment. Sleep duration was assessed with a single open-ended question about the number of hours participants usually sleep each night, expressed as minutes slept within 24 hours. The variable was categorized into three groups: recommended sleep duration (7–8 hours/day), short sleep duration (< 7 hours/day) and long sleep duration (> 8 hours/day). These categories are based on the recommended sleep duration for adults aged 65 years and above [39].

### Statistical methods

Statistical analyses were performed using SPSS Statistics (version 28, IBM Corp, Armonk, NY, USA) and Stata Statistical Software (release 17, StataCorp LLC, College Station, Texas, USA). Descriptive statistics were used to describe sample characteristics at baseline. Continuous variables were presented as means ± SD (if normally distributed) or as medians with IQR (if not normally distributed). Categorical variables were presented as frequencies and percentages. Recognizing the importance of considering sex differences in health research [40, 41], we consistently tested for effect modification by sex across the main analyses. This was achieved by adding an interaction term to the crude models (adjusted for age only) and evaluating statistical significance (*P* < .05). If a statistically significant interaction was found, models were stratified by sex. We additionally investigated whether the presence of caffeine in coffee moderates the association between coffee consumption and frailty status. To assess this, we added interaction terms between coffee consumption and the type of coffee consumed (caffeinated only vs. decaffeinated only) to the crude models (adjusted for age and sex) and evaluated statistical significance (*P* < .05). In doing so, non-coffee consumers and those who consumed both caffeinated and decaffeinated coffee were excluded. Models were finally adjusted for relevant confounders. These included age, sex (if not an effect modifier), education, partner status, smoking status, alcohol use, tea consumption, BMI, number of chronic diseases, depressive symptoms, cognitive function, and sleep duration. For analyses involving individual frailty components, additional adjustments were made for the remaining frailty components.

### Associations between habitual coffee consumption and the prevalence of frailty, pre-frailty, and frailty components

Associations between habitual coffee consumption and the prevalence of frailty, pre-frailty and individual components were examined using pooled data collected in 2018/2019 (coffee consumption and frailty) and in 2021/2022 (coffee consumption and frailty). Logistic Generalized Estimating Equation (GEE) models were used to calculate odds ratios (ORs) and confidence intervals (CI) for associations of habitual coffee consumption with (1) frailty (robust vs. frail), (2) pre-frailty (robust vs. pre-frail), and (3) individual underlying components of frailty (i.e., weight loss, weakness, exhaustion, slow gait speed, and low physical activity). GEE is a population-average approach that accounts for repeated measures within individuals [42]. Effect estimates from a logistic GEE are expected to be more precise than those obtained from logistic mixed model analyses [43, 44]. To minimize the potential for variation in frailty status across time points, we applied the 2018/2019 cut-off values—to establish the lowest quintile of gait speed and physical activity—to determine frailty status at 2021/2022. We excluded observations with missing data on coffee consumption, frailty status, and relevant confounders (< 5% missing data), resulting in a total of 2087 observations. Observations categorized as pre-frail (n Obs.=925) were excluded from the analyses with frailty (robust vs. frail), and observations categorized as frail (n Obs.=154) were excluded from the analyses with pre-frailty (robust vs. pre-frail) (Supplementary Figure S1). Observations with missing values on any of the components (n Obs.=147) were excluded from the analyses with individual frailty components.

### Associations between habitual coffee consumption and the incidence of frailty, pre-frailty or frailty, and frailty components

Associations between habitual coffee consumption and the incidence of (pre-)frailty with a 3-year follow-up were examined using data collected in 2018/2019 (coffee consumption and frailty) and in 2021/2022 (frailty). Analyses with a 7-year follow-up were examined using data collected in 2014/2015 (coffee consumption), 2015/2016 (frailty), 2018/2019 (frailty), and 2021/2022 (frailty). Cox proportional hazards models were used to calculate hazard ratios (HR) and confidence intervals (CI) for the association between coffee consumption and the 3- and 7-year incidence of (1) frailty, (2) pre-frailty or frailty, and (3) individual underlying components of frailty. Despite having only two measurement points over the 3-year follow-up period, we opted for Cox proportional hazards analysis to maintain consistency with the analyses conducted over a 7-year follow-up. In both analyses, we used the baseline cut-off values to determine frailty status at the follow-up measurements. The baseline for the 3-year incidence of frailty was 2018/2019, and the baseline for the 7-year incidence of frailty was 2015/2016. Respondents with missing data on coffee consumption and relevant confounders (< 5% missing data) were excluded. Individuals who were frail at baseline were excluded from the analyses of frailty incidence, while those who were frail or pre-frail at baseline were excluded from the analyses of pre-frailty or frailty incidence. The same approach was taken for analyses with individual underlying components of frailty. For instance, when analyzing the underlying frailty component ‘weight loss’, we excluded respondents with weight loss at baseline. The sample for the 3-year frailty incidence consisted of 868 participants (of which 378 were pre-frail), and the sample for the 7-year frailty incidence consisted of 967 participants (of which 496 were pre-frail) (Supplementary Figure S2 and S3).

### Associations between retrospectively assessed midlife coffee consumption and the prevalence of frailty, pre-frailty, and frailty components in later life

Associations of retrospectively assessed midlife (ages 40–65) coffee consumption with frailty status and underlying components were examined using data collected in 2018/2019 (midlife coffee consumption and frailty) and in 2021/2022 (frailty). Logistic regression analyses were performed to calculate odds ratios (ORs) and confidence intervals (CI) for associations of midlife coffee consumption with (1) frailty (robust vs. frail), (2) pre-frailty (robust vs. pre-frail), and (3) individual underlying components of frailty. Frailty status was determined with data collected in 2018/2019 and 2021/2022, using the 2018/2019 cut-offs to determine frailty status in 2021/2022. Individuals who were frail at one time point and robust or pre-frail at the other time point were classified as ‘frail’. Those who were pre-frail (but not frail) at either time point were classified as ‘pre-frail’. Participants with missing data on midlife coffee consumption, frailty status, and relevant confounders (< 5% missing data) were excluded. The total sample included 1140 respondents. Pre-frail participants (*n* = 641) were excluded from the analyses with frailty (robust vs. frail), and frail participants (*n* = 126) were excluded from the analyses with pre-frailty (robust vs. pre-frail) (Supplementary Figure S4). In analyses with individual frailty components, participants with missing values on any of the components were also excluded (*n* = 29).

## Results

### Sample characteristics

The characteristics of the community-dwelling older adults included in this study are presented in Table 1. The characteristics are presented separately for each type of analyses because inclusion and exclusion criteria varied, resulting in different sub-samples. Females were slightly more represented across all sub-samples (50.4 to 52.6%). The mean ± SD age of the participants ranged from 69.4 ± 7.1 years at the 7-year baseline (2015/2016) to 72.7 ± 7.3 years in 2018/2019. The prevalence of pre-frailty and frailty in 2018/2019 was 42.4% and 7.0%, respectively. Of the 868 participants included in the 3-year follow-up, 490 (56.5%) were robust and 378 (43.5%) were pre-frail at baseline (2018/2019). Of the 967 participants included in the 7-year follow-up, 471 (48.7%) were robust and 496 (51.3%) were pre-frail at baseline (2015/2016). The majority of participants consumed between two and four cups of coffee on average per day. Non-coffee consumers were least represented (< 5%). Over two-thirds of coffee consumers reported consumption of caffeinated coffee only, while the remainder consumed only decaffeinated coffee or a combination of the two.

**Table 1:** Sample characteristics of the community-dwelling older adults from the Longitudinal Aging Study Amsterdam by type of analysis

	Prevalence of (pre-)frailty	Incidence of frailty	
	2018/2019^a^ (*n* = 1161)	3-year^b^ (*n* = 868)	7-year^c^ (*n* = 967)
Coffee consumption (cups/day) ^d^, mean ± SD	4.7 ± 2.7	4.8 ± 2.8	3.5 ± 1.9
Habitual coffee consumption ^d^, *n* (%)			
0 cups/day > 0–2 cups/day > 2–4 cups/day > 4–6 cups/day > 6 cups/day	37 (3.2) 149 (12.8) 426 (36.7) 264 (22.7) 285 (24.5)	29 (3.3) 100 (11.5) 312 (35.9) 203 (23.4) 224 (25.8)	41 (4.2) 256 (26.5) 443 (45.8) 177 (18.3) 50 (5.2)
Midlife coffee consumption ^d^, *n* (%)			
0 cups/day > 0–2 cups/day > 2–4 cups/day > 4–6 cups/day > 6 cups/day	25 (2.2) 90 (7.8) 349 (30.1) 274 (23.6) 402 (34.6)	NA NA NA NA NA	NA NA NA NA NA
Type of coffee, *n* (%)			
Only caffeinated Only decaffeinated Both caffeinated and decaffeinated	872 (75.1) 146 (12.6) 106 (9.1)	658 (75.8) 100 (11.5) 81 (9.3)	NA NA NA
Age in years, mean ± SD	72.7 ± 7.3	71.6 ± 6.6	69.4 ± 7.1
Female, *n* (%)	611 (52.6)	442 (50.9)	487 (50.4)
Educational level, *n* (%)			
Low Intermediate High	349 (30.1) 488 (38.6) 364 (31.4)	240 (27.6) 337 (38.8) 291 (33.5)	274 (28.3) 375 (38.8) 318 (32.9)
Living alone, *n* (%)	374 (32.2)	252 (29.0)	255 (26.4)
Smoking status, *n* (%)			
Never smoked Former smoker Current smoker	279 (24.0) 780 (67.2) 102 (8.8)	206 (23.7) 588 (67.7) 74 (8.5)	258 (26.7) 607 (62.8) 102 (10.5)
Alcohol use, *n* (%)			
No use Moderate use Above moderate use	151 (13.0) 891 (76.7) 119 (10.2)	81 (9.3) 691 (79.6) 96 (11.1)	110 (11.4) 736 (76.1) 121 (12.5)
Body Mass Index (kg/m2), mean ± SD	27.3 ± 7.3	27.0 ± 4.3	26.9 ± 4.2
Number of chronic diseases, mean ± SD	1.4 ± 1.1	1.3 ± 1.1	1.2 ± 1.0
Depressive symptoms (CES-D scale score ≥ 16), *n* (%)	137 (11.8)	72 (8.3)	64 (6.6)
Cognitive impairment (MMSE score ≤ 23), *n* (%)	32 (2.8)	16 (1.8)	17 (1.8)
Sleep duration, *n* (%)			
Recommended (7–8 h/night) Short (< 7 h/night) Long (> 8 h/night)	745 (64.2) 252 (21.7) 164 (14.1)	587 (67.6) 173 (19.9) 108 (12.4)	633 (65.5) 191 (19.8) 143 (14.8)
Tea consumption, *n* (%)			
0 cups/day > 0–2 cups/day > 2–4 cups/day > 4 cups/day Missings	94 (8.1) 275 (23.7) 316 (27.2) 348 (30.0) 128 (11.0)	77 (8.9) 209 (24.1) 232 (26.7) 254 (29.3) 96 (11.1)	129 (13.3) 483 (49.9) 244 (25.2) 111 (11.5) 0 (0)
Frailty status, *n* (%)			
Robust Pre-frail Frail	558 (50.6) 492 (42.4) 81 (7.0)	490 (56.5) 378 (43.5) NA^e^	471 (48.7) 496 (51.3) NA^e^
Frailty components fulfilled ^f^, *n* (%)			
Weight loss Low grip strength Exhaustion Low physical activity Slow gait speed	153 (13.2) 228 (19.6) 104 (9.0) 213 (18.3) 206 (17.7)	88 (10.1) 122 (14.1) 45 (5.2) 112 (12.9) 112 (12.9)	173 (17.9) 190 (19.6) 39 (4.0) 105 (10.9) 132 (13.7)

Abbreviations: n = number of participants, SD = Standard Deviation, NA = Not Applicable

^a^ Baseline characteristics of participants included in the analyses of habitual coffee consumption and (pre-)frailty (*n* = 1161), as well as midlife coffee consumption and (pre-)frailty (*n* = 1140)

^b^ 2018/2019 baseline characteristics of the robust and pre-frail community-dwelling older adults, included in the analyses with a 3-year follow-up

^c^ 2015/2016 baseline characteristics of the robust and pre-frail community-dwelling older adults, included in the analyses with a 7-year follow-up

^d^ In the prevalence and 3-year incidence analyses of (pre-)frailty, coffee consumption (habitual and midlife) was measured by a self-administered questionnaire. In the 7-year incidence analyses, coffee consumption was measured by a food frequency questionnaire

^e^ Participants with frailty at baseline were excluded from the analyses

^f^ The number of participants varies due to missings. The percentage given is the proportion of the total

### Associations between habitual coffee consumption and the prevalence of frailty, pre-frailty, and frailty components

Associations of habitual coffee consumption with frailty and pre-frailty are presented in Table 2. In the fully adjusted model, the odds (95%CI) of frailty were 0.36 (0.16–0.82) times lower for those who consumed > 4–6 cups of coffee per day, and 0.37 (0.16–0.84) times lower for those who consumed > 6 cups/day, compared to the reference group (> 0–2 cups/day). The odds of pre-frailty were 0.73 (0.54–0.99) times lower for those who consumed > 2–4 cups/day, compared to the reference group. Analyses of individual frailty components showed that, after adjusting for confounders, consuming > 2–4 cups of coffee per day was associated with a 0.60 (0.40–0.90) lower odds of weight loss compared to the reference group (Supplementary Table S1). Moreover, consumption of > 2–4 cups, > 4–6 cups, and > 6 cups per day was associated with lower odds of weakness measured by grip strength, with ORs (95%CI) of 0.69 (0.48–0.98), 0.60 (0.41–0.89), and 0.55 (0.37–0.82), respectively (Supplementary Table S1).

**Table 2:** Associations between habitual coffee consumption and the prevalence of frailty (robust vs. frail) and pre-frailty (robust vs. pre-frail) in community-dwelling older adults of the Longitudinal Aging Study Amsterdam (n Obs.=2087)

Model	Frailty		Pre-frailty	
	OR (95%CI)	*P*-value	OR (95%CI)	*P*-value
**Habitual coffee consumption**				
Crude model*				
0 cups/day	0.90 (0.27–3.06)	0.868	0.97 (0.57–1.65)	0.923
>0–2 cups/day	Ref.	-	Ref.	-
>2–4 cups/day	0.42 (0.24–0.73)	**0.002**	0.70 (0.52–0.93)	**0.016**
>4–6 cups/day	0.28 (0.15–0.53)	**0.000**	0.84 (0.61–1.15)	0.268
>6 cups/day	0.37 (0.19–0.72)	**0.003**	0.87 (0.63–1.21)	0.405
*Adjusted model* **				
0 cups/day	1.05 (0.30–3.68)	0.940	0.97 (0.57–1.66)	0.926
>0–2 cups/day	Ref.	-	Ref.	-
>2–4 cups/day	0.53 (0.25–1.10)	0.087	0.73 (0.54–0.99)	**0.044**
>4–6 cups/day	0.36 (0.16–0.82)	**0.015**	0.84 (0.61–1.17)	0.314
>6 cups/day	0.37 (0.16–0.84)	**0.018**	0.80 (0.57–1.13)	0.203

Abbreviations: *n* Obs.=number of observations, OR = Odds Ratio, CI = Confidence Interval, Ref.=reference group

*Adjusted for sex and age, **Adjusted for sex, age, education, partner status, smoking status, alcohol use, tea consumption, body mass index, number of chronic diseases, depressive symptoms, cognitive function, and sleep duration

### Associations between habitual coffee consumption and the incidence of frailty, pre-frailty or frailty, and frailty components

Tables 3 and 4 show the longitudinal associations between habitual coffee consumption and the incidence of (pre-)frailty over 3- and 7 years, respectively. During the 3-year follow-up, 53 (6.1%) of the robust or pre-frail participants developed frailty, and 210 (42.9%) of the robust participants developed either frailty or pre-frailty. During the 7-year follow-up, 79 (8.2%) of the robust or pre-frail participants developed frailty, and 255 (54.1%) of the robust participants developed either frailty or pre-frailty. No statistically significant associations were found between habitual coffee consumption and the 3- and 7-year incidence of (pre)frailty and individual frailty components, except for a 59% lower risk (HR: 0.41 [95%CI 0.23–0.71]) of frailty after 7 years for the consumption of > 2–4 cups/day compared to the reference group (> 0–2 cups/day) (Tables 3 and 4 and supplementary Tables S2 and S3).

**Table 3:** Associations between habitual coffee consumption and the 3-year incidence of frailty (in robust or pre-frail individuals), and pre-frailty or frailty (in robust individuals) in community-dwelling older adults of the Longitudinal Aging Study Amsterdam

Model	Frailty (*n* = 868)^a^		Pre-frailty or frailty (*n* = 490)^b^	
	HR (95%CI)	*P*-value	HR (95%CI)	*P*-value
**Habitual coffee consumption**				
Crude model*				
0 cups/day	1.62 (0.45–5.92)	0.462	0.88 (0.34–2.33)	0.802
>0–2 cups/day	Ref.	-	Ref.	-
>2–4 cups/day	0.73 (0.29–1.86)	0.507	0.92 (0.58–1.46)	0.730
>4–6 cups/day	0.63 (0.22–1.85)	0.401	0.95 (0.57–1.57)	0.839
>6 cups/day	1.20 (0.46–3.11)	0.710	0.95 (0.58–1.55)	0.835
Adjusted model**				
0 cups/day	0.89 (0.20–4.02)	0.877	1.31 (0.47–3.64)	0.604
>0–2 cups/day	Ref.	-	Ref.	-
>2–4 cups/day	0.65 (0.24–1.77)	0.399	0.92 (0.57–1.48)	0.723
>4–6 cups/day	0.54 (0.17–1.69)	0.291	1.01 (0.60–1.70)	0.982
>6 cups/day	0.79 (0.29–2.14)	0.636	0.90 (0.54–1.48)	0.672

Abbreviations: HR = Hazard Ratio, CI = Confidence Interval, Ref.=reference group

^a^ Total number of events: 53/868, ^b^ Total number of events: 210/490

*Adjusted for sex and age **Adjusted for sex, age, education, partner status, smoking status, alcohol use, tea consumption, body mass index, number of chronic diseases, depressive symptoms, cognitive function, and sleep duration

**Table 4:** Associations between habitual coffee consumption and the 7-year incidence of frailty (in robust or pre-frail individuals), and pre-frailty or frailty (in robust individuals) in community-dwelling older adults of the Longitudinal Aging Study Amsterdam

Model	Frailty (*n* = 967)^a^		Pre-frailty or frailty (*n* = 471)^b^	
	HR (95%CI)	*P*-value	HR (95%CI)	*P*-value
**Habitual coffee consumption**				
Crude model*				
0 cups/day	1.56 (0.60–4.07)	0.366	0.73 (0.33–1.61)	0.432
>0–2 cups/day	Ref.	-	Ref.	-
>2–4 cups/day	0.49 (0.29–0.82)	**0.007**	1.03 (0.75–1.40)	0.879
>4–6 cups/day	0.74 (0.38–1.44)	0.368	1.31 (0.91–1.88)	0.141
>6 cups/day	0.84 (0.25–2.81)	0.779	1.60 (0.94–2.72)	0.082
Adjusted model**				
0 cups/day	1.16 (0.41–3.28)	0.774	0.83 (0.36–1.92)	0.664
>0–2 cups/day	Ref.	-	Ref.	-
>2–4 cups/day	0.41 (0.23–0.71)	**0.001**	0.98 (0.70–1.36)	0.879
>4–6 cups/day	0.64 (0.31–1.31)	0.221	1.21 (0.83–1.77)	0.325
>6 cups/day	0.52 (0.15–1.87)	0.317	1.27 (0.70–2.29)	0.434

Abbreviations: HR = Hazard Ratio, CI = Confidence Interval, Ref.=reference group

^a^ Total number of events: 79/967, ^b^ Total number of events: 255/471

*Adjusted for sex and age **Adjusted for sex, age, education, partner status, smoking status, alcohol use, tea consumption, body mass index, number of chronic diseases, depressive symptoms, cognitive function, and sleep duration

### Association between retrospectively assessed midlife coffee consumption and the prevalence of frailty, pre-frailty, and frailty components in later life

Associations between retrospectively assessed coffee consumption during midlife (ages 40–65) and the prevalence of frailty and pre-frailty are presented in Table 5. Midlife coffee consumption was not associated with frailty, pre-frailty, or individual frailty components in later life, with the exception of a lower odds of the frailty component “slow gait speed” for participants who consumed > 6 cups/day compared with those who consumed > 0–2 cups/day (OR 0.53 [95%CI 0.28-1.00]) (Table 5 and Supplementary Table S4).

**Table 5:** Associations between midlife coffee consumption and the prevalence of frailty (robust vs. frail) and pre-frailty (robust vs. pre-frail) in later life among community-dwelling older adults (*n* = 1140) of the Longitudinal Aging Study Amsterdam

Model	Frailty		Pre-frailty	
	OR (95%CI)	*P*-value	OR (95%CI)	*P*-value
**Midlife coffee consumption**				
Crude model*				
0 cups/day	0.79 (0.13–4.71)	0.797	0.98 (0.36–2.67)	0.964
>0–2 cups/day	Ref.	-	Ref.	-
>2–4 cups/day	0.48 (0.20–1.14)	0.096	0.76 (0.44–1.29)	0.308
>4–6 cups/day	0.47 (0.19–1.15)	0.098	0.88 (0.51–1.53)	0.648
>6 cups/day	0.53 (0.22–1.24)	0.143	1.06 (0.62–1.82)	0.852
*Adjusted model* **				
0 cups/day	1.44 (0.11–18.72)	0.781	0.96 (0.34–2.72)	0.934
>0–2 cups/day	Ref.	-	Ref.	-
>2–4 cups/day	0.55 (0.18–1.71)	0.299	0.73 (0.42–1.27)	0.262
>4–6 cups/day	0.52 (1.16–1.72)	0.286	0.84 (0.47–1.48)	0.538
>6 cups/day	0.44 (0.14–1.40)	0.164	0.89 (0.50–1.57)	0.684

Abbreviations: OR = Odds Ratio, CI = Confidence Interval, Ref.=reference group

*Adjusted for sex and age, **Adjusted for sex, age, education, partner status, smoking status, alcohol use, tea consumption, body mass index, number of chronic diseases, depressive symptoms, cognitive function, and sleep duration

### Effect modification by sex and coffee type

Neither sex nor coffee type (caffeinated only vs. decaffeinated only) were effect modifiers in the associations between habitual and midlife coffee consumption and the prevalence of frailty or pre-frailty (*P* >.05). Sex was also not an effect modifier in longitudinal associations with a 7-year follow-up (*P* >.05). In the analyses of the 3-year incidence of pre-frailty or frailty, there was a statistically significant interaction between categories of coffee consumption and sex (*P* interaction = 0.047 for > 6 cups/day vs. the reference group). Stratified results of both frailty and pre-frailty or frailty incidence showed a difference in the direction of associations between sexes. However, none of these associations were statistically significant in both crude and adjusted models (data not shown). Effect modification by coffee type was also observed in the analyses of pre-frailty or frailty incidence with a 3-year follow-up (*P*-value interaction = 0.044 for > 2–4 cups/day vs. the reference group). In the stratified and fully adjusted model, consumption of > 2–4 cups of decaffeinated coffee per day was associated with a 92% lower hazard (HR 0.08 [95%CI 0.01–0.47]) of pre-frailty or frailty incidence, compared to consuming > 0–2 cups/day (supplementary Table S5). Caffeinated coffee consumption was not associated with pre-frailty or frailty incidence (supplementary Table S5).

## Discussion

In this study of community-dwelling older adults participating in the Longitudinal Aging Study Amsterdam, habitual coffee consumption of > 4–6 cups (1 cup = 125 mL) and > 6 cups per day was associated with lower odds of frailty compared with consumption of > 0–2 cups/day. The associations between retrospectively assessed coffee consumption during midlife (ages 40–65) and frailty status, and between habitual coffee consumption and the incidence of frailty over three and seven years were of similar magnitude but did not achieve statistical significance, except for a 59% lower hazard (HR: 0.41 [95%CI 0.23–0.71]) of frailty after 7 years for those who consumed > 2–4 cups/day compared with those who consumed > 0–2 cups/day. No associations were found between coffee consumption and pre-frailty, with the exception of lower odds for those who consumed > 2–4 cups/day compared to > 0–2 cups/day (OR 0.73 [95%CI 0.54–0.99]).

Despite methodological differences and variations in previous study samples, the findings of our study are largely consistent with those of previous cross-sectional studies that observed an inverse association between coffee consumption and frailty [15–17]. Notable differences between our study and these prior studies are the inclusion of females only in the study by Kobayashi et al. [16], and the use of the frailty index instead of the frailty phenotype by Pang et al. [17]. While the present study showed a statistically significant association between habitual coffee consumption and 7-year incidence of frailty, a previous longitudinal study with a 7.2-year follow-up did not [19]. This previous study adjusted for a relatively large number of confounders, including physical activity, time spent watching television, energy intake, and a score for adherence to the Mediterranean diet. To ascertain whether the discrepancy in results was due to the different adjustments, we performed a sensitivity analysis in which we additionally adjusted for total energy intake (in Kilocalories) and a Mediterranean diet score, obtained from the FFQ data. The results of this sensitivity analysis did not differ in terms of statistical significance or effect size (data not shown). We did not adjust for physical activity in the present study to minimize the risk of over-adjustment, given the possible correlation between physical activity and frailty. The results of our study are also not fully consistent, regarding statistical significance, with those of an earlier longitudinal study by Chua et al. [18], which found that the consumption of ≥ 4 cups of coffee per day (equivalent to ≥ 7.5 cups of 125 mL in our study) at midlife was associated with reduced odds of physical frailty in later life (OR 0.54 [95% CI 0.38–0.76]). Nevertheless, the magnitude of the associations between midlife coffee consumption and frailty status observed in our study was comparable to that reported in the study by Chua et al. [18], which had a much greater sample size. This suggests that a lack of statistical power might explain the non-significance of our results, rather than a true absence of an association. It is also important to note that the assessment of coffee consumption during midlife was retrospective in the present study, representing a less comprehensive approach than that employed by Chua and colleagues [18], which utilized a longitudinal design with a 20-year follow-up.

Stratification of the results by type of coffee (decaffeinated vs. caffeinated) showed that a higher consumption of decaffeinated coffee was associated with a lower hazard of pre-frailty or frailty after three years, while higher consumption of caffeinated coffee was not associated with pre-frailty or frailty incidence. In the longitudinal study by Machado-Fragua et al. [19], no differences in associations by coffee type were found. However, stratified analyses, although non-significant, showed lower hazards of frailty for categories of decaffeinated coffee consumption and higher hazards of frailty for categories of caffeinated coffee consumption, which is consistent with the findings of our study. In our study, associations with frailty incidence could, however, not be stratified by coffee type due to a lack of respondents within subgroups. In contrast to our findings and those of Machado-Fragua et al. [19], Pang et al. [17] observed an inverse association between caffeinated coffee consumption and frailty, while no association was observed for decaffeinated coffee consumption. One potential reason for this discrepancy in findings may be the investigation of alternate study populations (European vs. American), potentially influencing coffee consumption patterns or methods of preparation [45]. Coffee consumption is deeply ingrained in Dutch culture, predominantly featuring black filter coffee, which may differ from other countries [46]. Another noteworthy aspect of the study by Pang et al. [17] is the utilization of the frailty index as the outcome measure, which is based on the accumulation of age-related deficits across multiple health domains, including physical, psychological, and social factors [47, 48]. More studies are needed to ascertain whether the relationship between coffee consumption and frailty is dependent on the type of coffee consumed.

Although the exact mechanisms by which coffee consumption may influence frailty development remain unclear, several potential factors have been identified. First, coffee is rich in antioxidants, including caffeine, polyphenols, and other bioactive compounds [11] that may reduce oxidative stress and inflammation [12, 13], both of which are associated with frailty [14, 49]. Moreover, coffee consumption may reduce the progression of sarcopenia, an age-related condition associated with frailty [50]. A prior in vivo and in vitro study with aged mice showed that coffee consumption attenuated their decline in muscle weight and grip strength [51], thereby reducing the risk of sarcopenia. These results are consistent with the results of our analyses of individual frailty components, wherein higher categories of coffee consumption were mostly associated with a lower odds of weight loss and weakness (as measured by grip strength). In addition, polyphenols in coffee may induce autophagy, which is essential for mitochondrial renewal and the prevention of muscle damage, thereby contributing to muscle maintenance [52]. Finally, evidence indicates that coffee consumption may improve insulin sensitivity and glucose uptake in muscles [53]. In summary, coffee consumption may potentially reduce the risk of physical frailty by delaying age-related sarcopenia and improving muscle integrity.

Strengths of this study include a comprehensive assessment of the relationship between coffee consumption and frailty status, considering both prevalence and incidence. Moreover, this study was the first to analyze associations between coffee consumption and individual underlying components of frailty, investigating potential underlying pathways. Furthermore, frailty status was consistently and systematically assessed using comprehensive and standardized methods at multiple time points. Our study also included a detailed assessment of coffee consumption in 2018/2019 and 2021/2022 (i.e., cup volume, type of coffee consumed, and midlife consumption). The FFQ data from 2014/2015 provided less detailed information on coffee consumption but allowed for the analysis of associations with (pre-)frailty incidence over a relatively long follow-up of seven years. Another strength of this study is the use of a nationally representative sample of community-dwelling older adults, which increases the generalizability of the findings. Some limitations of this study must also be considered. Despite adjusting for relevant confounding variables in the analyses, residual confounding may still be present. Moreover, potential measurement error in the assessment of coffee consumption might bias the observed associations. However, any error in the assessment of coffee consumption is likely to affect all participants equally. In the case of such non-differential measurement bias, associations may have been attenuated. The potential for recall bias is considered to be low, as habitual or frequently consumed foods, such as coffee, are remembered more accurately than foods consumed less frequently or without a pattern [54, 55]. Nevertheless, the potential is somewhat higher in analyses including retrospectively assessed coffee consumption during midlife. Given the study design and availability of data, it was not possible to obtain biological samples (e.g., blood or urine) from participants to validate self-reported coffee intake objectively or to examine biomarkers related to individual metabolic variations or polyphenol presence. Future studies incorporating biomarker analyses alongside self-reported data could provide more robust and comprehensive insights into the relationship between coffee consumption and frailty. Furthermore, our study may have lacked statistical power, potentially attributed to the low prevalence and incidence (pre-)frailty within our sample. Finally, it is important to note that the observational nature of the study precludes the ability to ascertain a cause-and-effect relationship between coffee consumption and (pre-)frailty.

In conclusion, the results of our study indicate that higher habitual coffee consumption is beneficially associated with frailty status in community-dwelling older adults. These findings suggest that habitual daily coffee consumption may contribute to improved health in older community-dwelling individuals. Nevertheless, further research is needed to confirm our findings, establish a possible causal relationship, and to identify the potential underlying mechanisms by which coffee and/ or coffee type might influence frailty development.

## Electronic supplementary material

Below is the link to the electronic supplementary material.

Supplementary Material 1

## Abbreviations

LASA: Longitudinal Aging Study Amsterdam
SaQ: Self-administered questionnaire
FFQ: Food frequency questionnaire
BMI: Body mass index
CES-D: Centre for epidemiologic studies depression scale
MMSE: Mini-mental state examination
GEE: Generalized estimating equations
HR: Hazard ratio
OR: Odds ratio
CI: Confidence interval

## References

[1] Fried LP, Tangen CM, Walston J et al (2001) Frailty in older adults: evidence for a phenotype. J Gerontol Biol Sci Med Sci 56:M146–M157. 10.1093/gerona/56.3.M146 10.1093/gerona/56.3.m146 11253156

[2] Dent E, Martin FC, Bergman H et al (2019) Management of frailty: opportunities, challenges, and future directions. Lancet 394:1376–1386. 10.1016/S0140-6736(19)31785-4 31609229 10.1016/S0140-6736(19)31785-4

[3] Kojima G, Iliffe S, Jivraj S, Walters K (2016) Association between frailty and quality of life among community-dwelling older people: a systematic review and meta-analysis. J Epidemiol Community Health 70:716–721. 10.1136/jech-2015-206717 26783304 10.1136/jech-2015-206717

[4] Kojima G, Taniguchi Y, Aoyama R, Tanabe M (2022) Associations between loneliness and physical frailty in community-dwelling older adults: A systematic review and meta-analysis. Ageing Res Rev 81:101705. 10.1016/j.arr.2022.101705 35932978 10.1016/j.arr.2022.101705

[5] Hoogendijk EO, Afilalo J, Ensrud KE et al (2019) Frailty: implications for clinical practice and public health. Lancet 394:1365–1375. 10.1016/S0140-6736(19)31786-6 31609228 10.1016/S0140-6736(19)31786-6

[6] Rashidi Pour Fard N, Amirabdollahian F, Haghighatdoost F (2019) Dietary patterns and frailty: a systematic review and meta-analysis. Nutr Rev 77:498–513. 10.1093/nutrit/nuz007 31038679 10.1093/nutrit/nuz007

[7] Lorenzo-López L, Maseda A, De Labra C et al (2017) Nutritional determinants of frailty in older adults: A systematic review. BMC Geriatr 17:108. 10.1186/s12877-017-0496-2 28506216 10.1186/s12877-017-0496-2 PMC5433026

[8] Manal B, Suzana S, Singh DKA (2015) NUTRITION AND FRAILTY: A REVIEW OF CLINICAL INTERVENTION STUDIES. J Frailty Aging 1–7. 10.14283/jfa.2015.49 10.14283/jfa.2015.49 27032052

[9] Poole R, Kennedy OJ, Roderick P et al (2017) Coffee consumption and health: umbrella review of meta-analyses of multiple health outcomes. BMJ 359:j5024. 10.1136/bmj.j5024 29167102 10.1136/bmj.j5024 PMC5696634

[10] Grosso G, Godos J, Galvano F, Giovannucci EL (2017) Coffee, caffeine, and health outcomes: an umbrella review. Annu Rev Nutr 37:131–156. 10.1146/annurev-nutr-071816-064941 28826374 10.1146/annurev-nutr-071816-064941

[11] Nuhu AA (2014) Bioactive micronutrients in coffee: recent analytical approaches for characterization and quantification. ISRN Nutr 2014:384230. 10.1155/2014/384230 24967266 10.1155/2014/384230 PMC4045301

[12] Rana A, Samtiya M, Dhewa T et al (2022) Health benefits of polyphenols: A concise review. J Food Biochem 46. 10.1111/jfbc.14264 10.1111/jfbc.14264 35694805

[13] Cory H, Passarelli S, Szeto J et al (2018) The role of polyphenols in human health and food systems: A Mini-Review. Front Nutr 5:87. 10.3389/fnut.2018.00087 30298133 10.3389/fnut.2018.00087 PMC6160559

[14] Moradi S, Hadi A, Mohammadi H et al (2021) Dietary inflammatory index and the risk of frailty among older adults: A systematic review and Meta-Analysis. Res Aging 43:323–331. 10.1177/0164027520948176 32815464 10.1177/0164027520948176

[15] De Nucci S, Zupo R, Donghia R et al (2023) Dietary profiling of physical frailty in older age phenotypes using a machine learning approach: the Salus in Apulia study. Eur J Nutr 62:1217–1229. 10.1007/s00394-022-03066-9 36484807 10.1007/s00394-022-03066-9 PMC10030526

[16] Kobayashi S, Asakura K, Suga H, Sasaki S (2014) Inverse association between dietary habits with high total antioxidant capacity and prevalence of frailty among elderly Japanese women: A multicenter cross-sectional study. J Nutr Health Aging 18:827–836. 10.1007/s12603-014-0556-725389961 10.1007/s12603-014-0478-4

[17] Pang S, Miao G, Zhou Y et al (2023) Association between coffee intake and frailty among older American adults: A population-based cross-sectional study. Front Nutr 10:1075817. 10.3389/fnut.2023.1075817 36819700 10.3389/fnut.2023.1075817 PMC9932698

[18] Chua KY, Li H, Lim W-S, Koh W-P (2023) Consumption of coffee, tea, and caffeine at midlife, and the risk of physical frailty in late life. J Am Med Dir. 10.1016/j.jamda.2023.06.015. Assoc S1525861023005753 10.1016/j.jamda.2023.06.015 37488031

[19] Machado-Fragua MD, Struijk EA, Graciani A et al (2019) Coffee consumption and risk of physical function impairment, frailty and disability in older adults. Eur J Nutr 58:1415–1427. 10.1007/s00394-018-1664-7 29549497 10.1007/s00394-018-1664-7

[20] Zhang N, Jia Z, Gu T et al (2023) Associations between modifiable risk factors and frailty: a Mendelian randomisation study. 10.1136/jech-2023-220882. J Epidemiol Community Health jech-2023-220882 10.1136/jech-2023-220882 37604674

[21] Huisman M, Poppelaars J, Van Der Horst M et al (2011) Cohort profile: the longitudinal aging study Amsterdam. Int J Epidemiol 40:868–876. 10.1093/ije/dyq219 21216744 10.1093/ije/dyq219

[22] Hoogendijk EO, Deeg DJH, Poppelaars J et al (2016) The longitudinal aging study Amsterdam: cohort update 2016 and major findings. Eur J Epidemiol 31:927–945. 10.1007/s10654-016-0192-0 27544533 10.1007/s10654-016-0192-0 PMC5010587

[23] Hoogendijk EO, Deeg DJH, De Breij S et al (2020) The longitudinal aging study Amsterdam: cohort update 2019 and additional data collections. Eur J Epidemiol 35:61–74. 10.1007/s10654-019-00541-2 31346890 10.1007/s10654-019-00541-2 PMC7058575

[24] Beukers MH, Dekker LH, De Boer EJ et al (2015) Development of the HELIUS food frequency questionnaires: ethnic-specific questionnaires to assess the diet of a multiethnic population in the Netherlands. Eur J Clin Nutr 69:579–584. 10.1038/ejcn.2014.180 25226823 10.1038/ejcn.2014.180

[25] Buta BJ, Walston JD, Godino JG et al (2016) Frailty assessment instruments: systematic characterization of the uses and contexts of highly-cited instruments. Ageing Res Rev 26:53–61. 10.1016/j.arr.2015.12.003 26674984 10.1016/j.arr.2015.12.003 PMC4806795

[26] Saum K, Müller H, Stegmaier C et al (2012) Development and evaluation of a modification of the fried frailty criteria using Population-Independent cutpoints. J Am Geriatr Soc 60:2110–2115. 10.1111/j.1532-5415.2012.04192.x 23043490 10.1111/j.1532-5415.2012.04192.x

[27] Hoogendijk EO, Van Hout HPJ, Heymans MW et al (2014) Explaining the association between educational level and frailty in older adults: results from a 13-year longitudinal study in the Netherlands. Ann Epidemiol 24:538–544e2. 10.1016/j.annepidem.2014.05.002 24935466 10.1016/j.annepidem.2014.05.002

[28] Hoogendijk EO, Suanet B, Dent E et al (2016) Adverse effects of frailty on social functioning in older adults: results from the longitudinal aging study Amsterdam. Maturitas 83:45–50. 10.1016/j.maturitas.2015.09.002 26428078 10.1016/j.maturitas.2015.09.002

[29] Stenholm S, Ferrucci L, Vahtera J et al (2019) Natural course of frailty components in people who develop frailty syndrome: evidence from two cohort studies. J Gerontol Biol Sci Med Sci 74:667–674. 10.1093/gerona/gly132 10.1093/gerona/gly132 PMC6477647 30084927

[30] Gruenewald TL, Seeman TE, Karlamangla AS, Sarkisian CA (2009) Allostatic load and frailty in older adults. J Am Geriatr Soc 57:1525–1531. 10.1111/j.1532-5415.2009.02389.x 19682116 10.1111/j.1532-5415.2009.02389.x PMC3650612

[31] Stel VS, Smit JH, Pluijm SMF et al (2004) Comparison of the LASA physical activity questionnaire with a 7-day diary and pedometer. J Clin Epidemiol 57:252–258. 10.1016/j.jclinepi.2003.07.008 15066685 10.1016/j.jclinepi.2003.07.008

[32] Stichting Voedingscentrum Nederland (2023) Maten En Gewichten. Nederlandse voedingsmiddelentabel, 52nd edn. Stichting Voedingscentrum Nederland, Den Haag, p 95

[33] Soroko S, Chang J, Barrett-Connor E (1996) Reasons for changing caffeinated coffee consumption: the rancho Bernardo study. J Am Coll Nutr 15:97–101. 10.1080/07315724.1996.10718571 8632123 10.1080/07315724.1996.10718571

[34] EFSA Panel on Dietetic Products, Nutrition and Allergies (NDA) (2015) Scientific opinion on the safety of caffeine. EFSA J 13. 10.2903/j.efsa.2015.4102

[35] Central Bureau of Statistics (1989) Health interview questionnaire. CBS, The Hague

[36] Radloff LS (1977) The CES-D scale: A Self-Report depression scale for research in the general population. Appl Psychol Meas 1:385–401. 10.1177/014662167700100306

[37] Beekman ATF, Deeg DJH, Van Limbeek J et al (1997) Criterion validity of the center for epidemiologic studies depression scale (CES-D): results from a community-based sample of older subjects in the Netherlands. Psychol Med 27:231–235. 10.1017/S0033291796003510 9122304 10.1017/s0033291796003510

[38] Berkman LF, Berkman CS, Kasl S et al (1986) Depressive symptoms in relation to physical health and functioning in the elderly. Am J Epidemiol 124:372–388. 10.1093/oxfordjournals.aje.a114408 3740038 10.1093/oxfordjournals.aje.a114408

[39] Hirshkowitz M, Whiton K, Albert SM et al (2015) National sleep foundation’s updated sleep duration recommendations: final report. Sleep Health 1:233–243. 10.1016/j.sleh.2015.10.004 29073398 10.1016/j.sleh.2015.10.004

[40] Gahagan J, Gray K, Whynacht A (2015) Sex and gender matter in health research: addressing health inequities in health research reporting. Int J Equity Health 14:12. 10.1186/s12939-015-0144-4 25637131 10.1186/s12939-015-0144-4 PMC4320818

[41] Mauvais-Jarvis F, Bairey Merz N, Barnes PJ et al (2020) Sex and gender: modifiers of health, disease, and medicine. Lancet 396:565–582. 10.1016/S0140-6736(20)31561-0 32828189 10.1016/S0140-6736(20)31561-0 PMC7440877

[42] Liang K-Y, Zeger SL (1986) Longitudinal data analysis using generalized linear models. Biometrika 73:13–22. 10.1093/biomet/73.1.13

[43] Twisk J, De Vente W, Apeldoorn A, De Boer MR (2022) Should we use logistic mixed model analysis for the effect Estimation in a longitudinal RCT with a dichotomous outcome variable? Epidemiol Biostat Public Health 14. 10.2427/12613

[44] Hubbard AE, Ahern J, Fleischer NL et al (2010) To GEE or not to GEE: comparing population average and mixed models for estimating the associations between neighborhood risk factors and health. Epidemiology 21:467–474. 10.1097/EDE.0b013e3181caeb90 20220526 10.1097/EDE.0b013e3181caeb90

[45] Landais E, Moskal A, Mullee A et al (2018) Coffee and tea consumption and the contribution of their added ingredients to total energy and nutrient intakes in 10 European countries: benchmark data from the late 1990s. Nutrients 10:725. 10.3390/nu10060725 29874819 10.3390/nu10060725 PMC6024313

[46] Centre for the Promotion of Imports from developing countries (2021) The Dutch market potential for coffee. In: Dutch Mark. Potential Coffee. https://www.cbi.eu/market-information/coffee/netherlands-0/market-potential. Accessed 28 Feb 2024

[47] Rockwood K (2005) A global clinical measure of fitness and frailty in elderly people. Can Med Assoc J 173:489–495. 10.1503/cmaj.050051 16129869 10.1503/cmaj.050051 PMC1188185

[48] Searle SD, Mitnitski A, Gahbauer EA et al (2008) A standard procedure for creating a frailty index. BMC Geriatr 8:24. 10.1186/1471-2318-8-24 18826625 10.1186/1471-2318-8-24 PMC2573877

[49] Soysal P, Isik AT, Carvalho AF et al (2017) Oxidative stress and frailty: A systematic review and synthesis of the best evidence. Maturitas 99:66–72. 10.1016/j.maturitas.2017.01.006 28364871 10.1016/j.maturitas.2017.01.006

[50] Wilson D, Jackson T, Sapey E, Lord JM (2017) Frailty and sarcopenia: the potential role of an aged immune system. Ageing Res Rev 36:1–10. 10.1016/j.arr.2017.01.006 28223244 10.1016/j.arr.2017.01.006

[51] Guo Y, Niu K, Okazaki T et al (2014) Coffee treatment prevents the progression of sarcopenia in aged mice in vivo and in vitro. Exp Gerontol 50:1–8. 10.1016/j.exger.2013.11.005 24269808 10.1016/j.exger.2013.11.005

[52] Pietrocola F, Malik SA, Mariño G et al (2014) Coffee induces autophagy in vivo. Cell Cycle 13:1987–1994. 10.4161/cc.28929 24769862 10.4161/cc.28929 PMC4111762

[53] Loureiro LMR, Reis CEG, Da Costa THM (2018) Effects of coffee components on muscle glycogen recovery: A systematic review. Int J Sport Nutr Exerc Metab 28:284–293. 10.1123/ijsnem.2017-0342 29345166 10.1123/ijsnem.2017-0342

[54] Ambrosini GL, Van Roosbroeck SAH, Mackerras D et al (2003) The reliability of Ten-Year dietary recall: implications for cancer research. J Nutr 133:2663–2668. 10.1093/jn/133.8.2663 12888655 10.1093/jn/133.8.2663

[55] Krall EA, Dwyer JT, Ann Coleman K (1988) Factors influencing accuracy of dietary recall. Nutr Res 8:829–841. 10.1016/S0271-5317(88)80162-3

