# Supplementary material for: Habitual coffee consumption and risk of frailty in later life: the Longitudinal Aging Study Amsterdam (LASA)
Source: Eur J Nutr. 2025 Apr 24;64(4):164. doi: 10.1007/s00394-025-03683-0 (PMC12021940; doi:10.1007/s00394-025-03683-0)
Supplement: Supplementary file 1 — Supplementary Material 1 [file 394_2025_3683_MOESM1_ESM.docx]

**Habitual coffee consumption and risk of frailty in later life: the Longitudinal Aging Study Amsterdam (LASA)**

Mette van der Linden^a^, Hanneke A.H. Wijnhoven^a^, Laura A. Schaap^a,b^, Emiel O. Hoogendijk^c,d^, Margreet R. Olthof^a^

^a^ Department of Health Sciences, Faculty of Science, Amsterdam Public Health Research Institute, Vrije Universiteit Amsterdam, Amsterdam, the Netherlands

^b^ Amsterdam Movement Sciences, Amsterdam, The Netherlands.

^c^ Department of Epidemiology & Data Science, Amsterdam Public Health Research Institute, Amsterdam UMC - location VU University Medical Center, Amsterdam, The Netherlands

^d^ Department of General Practice, Amsterdam Public Health Research Institute, Amsterdam UMC – location VU University Medical Center, Amsterdam, the Netherlands

Correspondence to: Mette van der linden; Department of Health Sciences, Faculty of Science, Vrije Universiteit Amsterdam, 1081 HV Amsterdam, The Netherlands; Email: [mettevdl@gmail.com](mailto:margreet.olthof@vu.nl); ORCID iD: <https://orcid.org/0009-0004-5859-9324>

**Supplementary Fig. S1** Flowchart of observations included in the analytic samples for associations of habitual coffee consumption with frailty (robust vs. frail) and pre-frailty (robust vs. pre-frail).

**Total number of observations LASA wave J (2018/2019) and K (2021/2022): n Obs.=3094**

Total study population wave J: n=1701

Total study population wave K: n=1393

**Valid data on coffee consumption wave J and K: n Obs.= 2202**

Valid data on coffee consumption wave J: n= 1221

Valid data on coffee consumption wave K: n= 981

**Valid data on coffee consumption and frailty status: n Obs.=2128**

Valid data on coffee consumption and known frailty status wave J: n=1184

Valid data on coffee consumption and known frailty status wave K: n=944

**Frailty status is unknown due to missing frailty criteria: n Obs.= 74**

Frailty status is unknown due to missing frailty criteria wave J: n= 37

Frailty status is unknown due to missing frailty criteria wave K: n= 37

**No or invalid data on coffee consumption: n Obs.=930**

No valid data on coffee consumption wave J: n=480

No valid data on coffee consumption wave K: n= 412

**No or invalid data on relevant confounding variables: n Obs.=42**

No or invalid data on relevant confounding variables wave J: n=24

*BMI (n=11), depression (n=1), sleep duration (n=12)*

No or invalid data on relevant confounding variables wave K: n=24

*BMI (n=14), alcohol consumption (n=1), sleep duration (n=3)*

**Pre-frail: n Obs.=925**

**Valid data on coffee consumption, frailty status and valid data on relevant confounding variables: n Obs.=2087**

**Analytical sample for analysis with frailty: n Obs.= 1162**

**Frail: n Obs.=154**

**Analytical sample for analysis with pre-frailty: n Obs.= 1933**

**Supplementary Fig. S2** Flowchart of participants included in the analytic samples for the 3-year incidence of frailty and the 3-year incidence of pre-frailty or frailty.

**Valid baseline data on coffee consumption, no baseline (pre-)frailty, and known frailty status at follow-up: n=490**

**No or invalid data on coffee consumption: n= 480**

**Valid baseline data on coffee consumption: n= 1221**

**Baseline frailty status is unknown due to missing frailty criteria: n= 37**

**Frail at baseline: n=88**

**No or invalid follow-up data on frailty: n=218**

**No or invalid data on relevant confounding variables: n=10**

BMI (n=7), sleep duration (n=3)

**Valid baseline data on coffee consumption and frailty status, no baseline frailty, known frailty status at follow-up, and valid data on relevant confounders: n=868**

**Pre-frail at baseline: n=378**

**Total study population at baseline (2018/2019): n=1701**

**Supplementary Fig. S3** Flowchart of participants included in the analytic samples for the 7-year incidence of frailty and the 7-year incidence of pre-frailty or frailty.

**Total study population at baseline (2015/2016): n=2024**

**No or invalid data on coffee consumption (FFQ): n= 725**

**Baseline frailty status is unknown due to missing frailty criteria: n= 31**

**Frail at baseline: n= 112**

**No or invalid follow-up data on frailty: n= 158**

**No or invalid data on relevant confounding variables: n=31**

BMI (n=4), alcohol consumption (n=1), sleep duration (n=26)

**Valid baseline data on coffee consumption (FFQ) and frailty status, no baseline frailty, known frailty status at follow-up, and valid data on relevant confounders: n= 967**

**Pre-frail at baseline: n= 496**

**Valid baseline data on coffee consumption (FFQ), no baseline (pre-)frailty, and known frailty status at follow-up: n= 471**

**Valid baseline data on coffee consumption (FFQ): n= 1299**

**Supplementary Fig. S4** Flowchart of participants included in the analytic samples for associations of midlife coffee consumption with frailty (robust vs. frail) and pre-frailty (robust vs. pre-frail) in later life.

**Total number of participants LASA wave J (2018/2019): n=1701**

**Additional cases with valid info on frailty status wave K (2021/2022): n=42**

**Valid data on coffee consumption wave J: n=1201**

**Valid data on coffee consumption and frailty status**: **n=1165**

**1162**

**Frailty status at wave J (2018/2019) or K (2021/2022) is unknown due to missing frailty criteria: n=36**

**No or invalid data on midlife coffee consumption: n= 542**

**No or invalid data on relevant confounding variables: n=25**

*BMI (n=11), depressive symptoms (n=1), sleep duration (n=14)*

**Pre-frail: n=641**

**Valid data on coffee consumption, frailty status and valid data on relevant confounding variables: n=1140**

**Analytical sample for analysis with frailty: n=499**

**Frail: n=126**

**Analytical sample for analysis with pre-frailty: n=1014**

**Supplementary Table S1:** Associations between habitual coffee consumption and the prevalence of frailty components in community-dwelling older adults of the Longitudinal Ageing Study Amsterdam (n Obs.=1940).

|  |  | **Frailty components** | | | | | | | | | |
| --- | --- | --- | --- | --- | --- | --- | --- | --- | --- | --- | --- |
|  |  | *Weight loss* | | *Weakness* | | *Exhaustion* | | *Low physical activity* | | *Slow gait speed* | |
| **Habitual coffee consumption** |  | OR (95%CI) | *P*-value | OR (95%CI) | *P*-value | OR (95%CI) | *P*-value | OR (95%CI) | *P*-value | OR (95%CI) | *P*-value |
|  |  |  |  |  |  |  |  |  |  |  |  |
| *Crude model** |  |  |  |  |  |  |  |  |  |  |  |
| 0 cups/day |  | 1.07 (0.57-2.01) | 0.829 | 0.88 (0.48-1.61) | 0.676 | 0.86 (0.35-2.10) | 0.743 | 0.88 (0.44-1.75) | 0.717 | 0.97 (0.47-2.01) | 0.942 |
| >0-2 cups/day |  | Ref. | - | Ref. | - | Ref. | - | Ref. | - | Ref. | - |
| >2-4 cups/day |  | 0.54 (0.36-0.80) | **0.002** | 0.67 (0.48-0.93) | **0.017** | 0.67 (0.40-1.10) | 0.115 | 0.83 (0.59-1.18) | 0.300 | 0.84 (0.58-1.21) | 0.353 |
| >4-6 cups/day |  | 0.82 (0.55-1.22) | 0.321 | 0.58 (0.40-0.84) | **0.004** | 0.66 (0.37-1.16) | 0.146 | 0.73 (0.49-1.08) | 0.117 | 0.64 (0.42-0.97) | **0.038** |
| >6 cups/day |  | 0.78 (0.51-1.18) | 0.233 | 0.59 (0.40-0.86) | **0.007** | 0.67 (0.37-1.23) | 0.199 | 0.82 (0.55-1.22) | 0.331 | 0.86 (0.57-1.30) | 0.476 |
| *Adjusted model* ** |  |  |  |  |  |  |  |  |  |  |  |
| 0 cups/day |  | 1.05 (0.54-2.03) | 0.882 | 0.84 (0.45-1.55) | 0.571 | 0.78 (0.23-2.57) | 0.679 | 0.90 (0.43-1.87) | 0.770 | 0.94 (0.44-2.00) | 0.874 |
| >0-2 cups/day |  | Ref. | - | Ref. | - | Ref. | - | Ref. | - | Ref. | - |
| >2-4 cups/day |  | 0.60 (0.40-0.90) | **0.014** | 0.69 (0.48-0.98) | **0.038** | 0.91 (0.49-1.66) | 0.752 | 0.92 (0.64-1.33) | 0.647 | 0.96 (0.65-1.43) | 0.848 |
| >4-6 cups/day |  | 0.96 (0.63-1.46) | 0.847 | 0.60 (0.41-0.89) | **0.010** | 0.80 (0.41-1.57) | 0.523 | 0.83 (0.55-1.26) | 0.382 | 0.70 (0.45-1.11) | 0.130 |
| >6 cups/day |  | 0.86 (0.56-1.34) | 0.510 | 0.55 (0.37-0.82) | **0.004** | 0.75 (0.36-1.55) | 0.436 | 0.82 (0.53-1.26) | 0.365 | 0.85 (0.55-1.31) | 0.457 |

Abbreviations: n Obs.= number of observations, OR= Odds Ratio, CI= Confidence Interval. Ref. = reference group

*Adjusted for sex and age, **Adjusted for sex, age, education, partner status, smoking status, alcohol use, tea consumption, body mass index, number of chronic diseases, depressive symptoms, cognitive function, sleep duration, and remaining frailty components.

**Supplementary Table S2**: Associations between habitual coffee consumption and the 3-year incidence frailty components in robust or pre-frail community-dwelling older adults of the Longitudinal Ageing Study Amsterdam.

|  |  | **Frailty components** | | | | | | | | | |
| --- | --- | --- | --- | --- | --- | --- | --- | --- | --- | --- | --- |
|  |  | *Weight loss (n=734)^a^* | | *Weakness (n=702)^b^* | | *Exhaustion (n=786)^c^* | | *Low physical activity (n=720)^d^* | | *Slow gait speed (n=707)^e^* | |
| **Habitual coffee consumption** |  | HR (95%CI) | *P*-value | HR (95%CI) | *P*-value | HR (95%CI) | *P*-value | HR (95%CI) | *P*-value | HR (95%CI) | *P*-value |
|  |  |  |  |  |  |  |  |  |  |  |  |
| *Crude model** |  |  |  |  |  |  |  |  |  |  |  |
| 0 cups/day |  | 1.21 (0.45-3.21) | 0.707 | 0.86 (0.24-3.05) | 0.818 | 0.91 (0.18-4.60) | 0.907 | 1.18 (0.32-4.38) | 0.802 | 1.01 (0.21-4.79) | 0.991 |
| >0-2 cups/day |  | Ref. | - | Ref. | - | Ref. | - | Ref. | - | Ref. | - |
| >2-4 cups/day |  | 0.77 (0.42-1.44) | 0.413 | 1.03 (0.55-1.92) | 0.932 | 0.69 (0.26-1.79) | 0.443 | 1.16 (0.56-2.42) | 0.688 | 0.76 (0.34-1.71) | 0.507 |
| >4-6 cups/day |  | 0.91 (0.46-1.79) | 0.789 | 1.11 (0.58-2.15) | 0.749 | 0.41 (0.13-1.36) | 0.147 | 0.99 (0.44-2.20) | 0.971 | 0.82 (0.35-1.95) | 0.660 |
| >6 cups/day |  | 0.70 (0.36-1.38) | 0.304 | 1.30 (0.68-2.48) | 0.426 | 0.73 (0.26-2.06) | 0.548 | 1.73 (0.83-3.62) | 0.145 | 0.84 (0.37-1.92) | 0.676 |
| *Adjusted model* ** |  |  |  |  |  |  |  |  |  |  |  |
| 0 cups/day |  | 1.93 (0.67-5.57) | 0.225 | 0.94 (0.25-3.51) | 0.930 | 0.24 (0.03-2.34) | 0.219 | 1.20 (0.31-4.66) | 0.790 | 0.75 (0.14-4.01) | 0.734 |
| >0-2 cups/day |  | Ref. | - | Ref. | - | Ref. | - | Ref. | - | Ref. | - |
| >2-4 cups/day |  | 0.78 (0.41-1.50) | 0.461 | 0.99 (0.52-1.89) | 0.983 | 0.80 (0.29-2.24) | 0.672 | 1.15 (0.53-2.48) | 0.725 | 0.87 (0.38-2.03) | 0.753 |
| >4-6 cups/day |  | 0.89 (0.44-1.77) | 0.730 | 1.07 (0.54-2.11) | 0.850 | 0.31 (0.09-1.14) | 0.077 | 1.02 (0.44-2.36) | 0.957 | 0.73 (0.29-1.80) | 0.488 |
| >6 cups/day |  | 0.70 (0.35-1.39) | 0.304 | 1.06 (0.54-2.06) | 0.874 | 0.42 (0.13-1.33) | 0.140 | 1.57 (0.73-3.41) | 0.251 | 0.67 (0.28-1.60) | 0.362 |

Abbreviations: HR= Hazard Ratio, CI= Confidence Interval, Ref. = reference group

^a^ Total number of events: 116/734, ^b^ Total number of events: 131/702, ^c^ Total number of events: 37/786, ^d^ Total number of events: 105/720, ^e^ Total number of events: 69/707

*Adjusted for sex and age **Adjusted for sex, age, education, partner status, smoking status, alcohol use, tea consumption, body mass index, number of chronic diseases, depressive symptoms, cognitive function, sleep duration, and remaining frailty components.

**Supplementary Table S3** : Associations between habitual coffee consumption and the 7-year incidence of frailty components in robust or pre-frail community-dwelling older adults of the Longitudinal Ageing Study Amsterdam.

|  |  | **Frailty components** | | | | | | | | | |
| --- | --- | --- | --- | --- | --- | --- | --- | --- | --- | --- | --- |
|  |  | *Weight loss (n=746)* | | *Weakness (n=743)* | | *Exhaustion (n=891)* | | *Low physical activity (n=832)* | | *Slow gait speed (n=791)* | |
| **Habitual coffee consumption** |  | HR (95%CI) | *P*-value | HR (95%CI) | *P*-value | HR (95%CI) | *P*-value | HR (95%CI) | *P*-value | HR (95%CI) | *P*-value |
|  |  |  |  |  |  |  |  |  |  |  |  |
| *Crude model** |  |  |  |  |  |  |  |  |  |  |  |
| 0 cups/day |  | 1.15 (0.60-2.20) | 0.679 | 0.84 (0.33-2.14) | 0.713 | 0.92 (0.27-3.07) | 0.889 | 1.36 (0.62-2.96) | 0.441 | 0.55 (0.20-1.52) | 0.247 |
| >0-2 cups/day |  | Ref. | - | Ref. | - | Ref. | - | Ref. | - | Ref. | - |
| >2-4 cups/day |  | 0.69 (0.49-0.96) | **0.027** | 0.78 (0.51-1.17) | 0.225 | 0.63 (0.37-1.09) | 0.096 | 0.82 (0.53-1.27) | 0.368 | 1.03 (0.75-1.42) | 0.855 |
| >4-6 cups/day |  | 0.80 (0.52-1.23) | 0.309 | 1.16 (0.72-1.87) | 0.537 | 1.26 (0.68-2.34) | 0.468 | 1.50 (0.92-2.45) | 0.103 | 0.83 (0.53-1.29) | 0.402 |
| >6 cups/day |  | 1.04 (0.54-1.99) | 0.909 | 1.74 (0.90-3.38) | 0.100 | 2.06 (0.87-4.87) | 0.102 | 1.61 (0.76-3.37) | 0.212 | 1.30 (0.67-2.50) | 0.436 |
| *Adjusted model* ** |  |  |  |  |  |  |  |  |  |  |  |
| 0 cups/day |  | 1.21 (0.60-2.45) | 0.592 | 0.97 (0.37-2.55) | 0.945 | 0.86 (0.24-3.06) | 0.814 | 1.18 (0.51-2.73) | 0.696 | 0.62 (0.22-1.77) | 0.374 |
| >0-2 cups/day |  | Ref. | - | Ref. | - | Ref. | - | Ref. | - | Ref. | - |
| >2-4 cups/day |  | 0.71 (0.50-1.01) | 0.054 | 0.74 (0.48-1.14) | 0.169 | 0.63 (0.35-1.13) | 0.124 | 0.77 (0.49-1.22) | 0.269 | 1.02 (0.73-1.43) | 0.904 |
| >4-6 cups/day |  | 0.86 (0.55-1.34) | 0.501 | 1.13 (0.69-1.86) | 0.632 | 1.46 (0.74-2.87) | 0.279 | 1.51 (0.90-2.51) | 0.116 | 0.81 (0.51-1.29) | 0.371 |
| >6 cups/day |  | 1.03 (0.51-2.06) | 0.946 | 1.58 (0.78-3.23) | 0.208 | 1.66 (0.63-4.36) | 0.304 | 1.41 (0.63-3.17) | 0.407 | 1.03 (0.51-2.08) | 0.937 |

Abbreviations: HR= Hazard Ratio, CI= Confidence Interval, Ref. = reference group

^a^ Total number of events: 195/746, ^b^ Total number of events: 140/743, ^c^ Total number of events: 80/891, ^d^ Total number of events: 133/832, ^e^ Total number of events: 202/791

*Adjusted for sex and age **Adjusted for sex, age, education, partner status, smoking status, alcohol use, tea consumption, body mass index, number of chronic diseases, depressive symptoms, cognitive function, sleep duration, and remaining frailty components.

**Supplementary Table S4:** Associations between midlife coffee consumption and the prevalence of frailty components in community-dwelling older adults of the Longitudinal Ageing Study Amsterdam (n=1111).

|  |  | **Frailty components** | | | | | | | | | |
| --- | --- | --- | --- | --- | --- | --- | --- | --- | --- | --- | --- |
|  |  | *Weight loss* | | *Weakness* | | *Exhaustion* | | *Low physical activity* | | *Slow gait speed* | |
| **Midlife coffee consumption** |  | OR (95%CI) | *P*-value | OR (95%CI) | *P*-value | OR (95%CI) | *P*-value | OR (95%CI) | P-value | OR (95%CI) | *P*-value |
|  |  |  |  |  |  |  |  |  |  |  |  |
| *Crude model** |  |  |  |  |  |  |  |  |  |  |  |
| 0 cups/day |  | 2.25 (0.87-5.81) | 0.096 | 1.14 (0.43-2.98) | 0.795 | 1.04 (0.30-3.60) | 0.945 | 0.81 (0.30-2.18) | 0.671 | 0.27 (0.07-1.05) | 0.059 |
| >0-2 cups/day |  | Ref. | - | Ref. | - | Ref. | - | Ref. | - | Ref. | - |
| >2-4 cups/day |  | 0.97 (0.56-1.69) | 0.909 | 0.68 (0.41-1.13) | 0.139 | 0.66 (0.34-1.29) | 0.221 | 0.72 (0.43-1.20) | 0.201 | 0.72 (0.41-1.26) | 0.246 |
| >4-6 cups/day |  | 1.04 (0.59-1.83) | 0.905 | 0.66 (0.39-1.12) | 0.123 | 0.77 (0.39-1.52) | 0.450 | 0.66 (0.39-1.12) | 0.124 | 0.59 (0.33-1.06) | 0.077 |
| >6 cups/day |  | 1.17 (0.67-2.02) | 0.585 | 0.75 (0.45-1.25) | 0.268 | 0.87 (0.45-1.68) | 0.673 | 0.86 (0.52-1.43) | 0.561 | 0.61 (0.35-1.07) | 0.084 |
| *Adjusted model* ** |  |  |  |  |  |  |  |  |  |  |  |
| 0 cups/day |  | 2.21 (0.82-5.96) | 0.119 | 1.15 (0.41-3.23) | 0.785 | 1.30 (0.28-5.98) | 0.736 | 0.83 (0.28-2.47) | 0.739 | 0.25 (0.06-1.14) | 0.074 |
| >0-2 cups/day |  | Ref. | - | Ref. | - | Ref. | - | Ref. | - | Ref. | - |
| >2-4 cups/day |  | 1.01 (0.57-1.79) | 0.963 | 0.71 (0.41-1.22) | 0.214 | 0.92 (0.40-2.08) | 0.835 | 0.79 (0.45-1.38) | 0.409 | 0.75 (0.40-1.39) | 0.357 |
| >4-6 cups/day |  | 1.09 (0.61-1.95) | 0.783 | 0.71 (0.41-1.25) | 0.238 | 0.92 (0.40-2.13) | 0.847 | 0.71 (0.40-1.26) | 0.245 | 0.60 (0.32-1.14) | 0.116 |
| >6 cups/day |  | 1.21 (0.68-2.14) | 0.524 | 0.70 (0.41-1.22) | 0.212 | 0.92 (0.40-2.12) | 0.846 | 0.85 (0.49-1.50) | 0.584 | 0.53 (0.28-1.00) | **0.048** |

Abbreviations: OR= Odds Ratio, CI= Confidence Interval, Ref. = reference group

*Adjusted for sex and age, **Adjusted for sex, age, education, partner status, smoking status, alcohol use, tea consumption, body mass index, number of chronic diseases, depressive symptoms, cognitive function, sleep duration, and remaining frailty components.

**Supplementary Table S5** : Associations between habitual coffee consumption and the 3-year incidence of pre-frailty or frailty in community-dwelling older adults of the Longitudinal Ageing Study Amsterdam, stratified by type of coffee consumption (caffeinated vs. decaffeinated).

| **Habitual coffee consumption** |  | **Pre-frailty or frailty** | | | | |
| --- | --- | --- | --- | --- | --- | --- |
|  |  | **Caffeinated coffee only (n=367) ^a^** | |  | **Decaffeinated coffee only (n=59) ^b^** | |
|  |  | HR (95%CI) | *P*-value |  | HR (95%CI) | *P*-value |
| *Crude model** |  |  |  |  |  |  |
| >0-2 cups/day |  | Ref. | - |  | Ref. | - |
| >2-4 cups/day |  | 1.08 (0.62-1.87) | 0.790 |  | 0.25 (0.08-0.76) | **0.015** |
| >4-6 cups/day |  | 1.13 (0.61-2.08) | 0.700 |  | 0.50 (0.16-1.59) | 0.240 |
| >6 cups/day |  | 1.26 (0.69-2.30) | 0.457 |  | 0.22 (0.06-0.89) | **0.033** |
| *Adjusted model*** |  |  |  |  |  |  |
| >0-2 cups/day |  | Ref. | - |  | Ref. | - |
| >2-4 cups/day |  | 1.12 (0.63-2.01) | 0.694 |  | 0.08 (0.01-0.47) | **0.006** |
| >4-6 cups/day |  | 1.23 (0.65-2.33) | 0.524 |  | 0.30 (0.05-1.91) | 0.200 |
| >6 cups/day |  | 1.11 (0.59-2.07) | 0.752 |  | 0.15 (0.02-1.34) | 0.090 |

Abbreviations: HR= Hazard Ratio, CI= Confidence Interval, Ref. = reference group

^a^ Total number of events: 146/367, ^b^ Total number of events: 34/59

*Adjusted for sex and age **Adjusted for sex, age, education, partner status, smoking status, alcohol use, tea consumption, body mass index, number of chronic diseases, depressive symptoms, cognitive function, and sleep duration.
